# “People play it down and tell me it can’t kill people, but I know people are dying each day”. Children’s health literacy relating to a global pandemic (COVID-19); an international cross sectional study

**DOI:** 10.1371/journal.pone.0246405

**Published:** 2021-02-10

**Authors:** Lucy Bray, Bernie Carter, Lucy Blake, Holly Saron, Jennifer A. Kirton, Fanny Robichaud, Marla Avila, Karen Ford, Begonya Nafria, Maria Forsner, Stefan Nilsson, Andrea Chelkowski, Andrea Middleton, Anna-Clara Rullander, Janet Mattsson, Joanne Protheroe

**Affiliations:** 1 Faculty of Health, Social Care and Medicine, Edge Hill University, Ormskirk, United Kingdom; 2 UQO, Département de Sciences Infirmières, Québec, Canada; 3 Botucatu Medical School—Unesp—Nursing Department, Sao Paulo, Brazil; 4 Centre for Education and Research—Nursing and Midwifery, Tasmanian Health Service South and University of Tasmania, Hobart, Australia; 5 Sant Joan de Déu Research Foundation, Barcelona, Spain; 6 Department of Nursing, Faculty of Medicine, Umeå University, Umeå, Sweden; 7 Institute of Health and Care Sciences and Centre for Person-Centred Care, Sahlgrenska Academy, University of Gothenburg, Gothenburg, Sweden; 8 Department of Health Sciences, Department of Learning, The Swedish Red Cross University College, Informatics, Management and Ethics, LIME, Karolinska Institutet, Stockholm, Sweden; 9 Keele Medical School, Faculty of Medicine and Health Sciences, Keele University, Staffordshire, United Kingdom; University of South Florida, UNITED STATES

## Abstract

The aim of this study was to examine aspects of children’s health literacy; the information sources they were accessing, their information preferences, their perceived understanding of and their reported information needs in relation to COVID-19. An online survey for children aged 7–12 years of age and parent/caregivers from the UK, Sweden, Brazil, Spain, Canada and Australia was conducted between 6^th^ of April and the 1^st^ of June 2020. The surveys included demographic questions and both closed and open questions focussing on access to and understanding of COVID-19 information. Descriptive statistics and qualitative content analysis procedures were conducted. The findings show that parents are the main source of information for children during the pandemic in most countries (89%, n = 347), except in Sweden where school was the main source of information. However, in many cases parents chose to shield, filter or adapt their child’s access to information about COVID-19, especially in relation to the death rates within each country. Despite this, children in this study reported knowing that COVID-19 was deadly and spreads quickly. This paper argues for a community rather than individual approach to addressing children’s health literacy needs during a pandemic.

## Introduction

The COVID-19 virus has spread throughout the world and was declared a pandemic by the World Health Organisation (WHO) on the 11^th^ March 2020. The pandemic has caused unprecedented upheaval to societies all over the world, and by the time this study closed (June 2020) there had been over 9 million cases worldwide [1]. In most countries people were asked to mitigate the transmission of the virus and save lives by adhering where possible, to guidance to work from home, stay at home and maintain social distancing or movement control. For many children this has meant there have been massive changes to their lives, with ‘home-schooling’ and staying in touch with friends and extended family via remote methods. UNICEF [2] notes that children might find it difficult to understand what they are seeing and hearing about the pandemic and can be particularly vulnerable to feelings of anxiety, stress and sadness.

With the COVID-19 pandemic, as in previous epidemics, it is important for children and adults to be able to access and understand health and public information in order to ensure that they adhere to infection control precautions [3]. A person’s ability to obtain, process and use information to make choices and decisions about their health is termed health literacy [4]. Health literacy is more than reading and writing health related information, but relates to familial, organisational and societal influences on a person’s ability to access, understand and use information to shape their health [5].

Social media, television and the internet has been filled with information about COVID-19, and this has been termed an ‘infodemic’ [6]. However, despite this, research has shown that adults have low levels of health literacy in relation to this virus with many being unable to identify symptoms of infection [7] or appreciate that the virus is contagious [8]. At the time of writing, no studies could be identified which had explored aspects of children’s health literacy (access to and understanding of information) in relation to the COVID-19 pandemic. However, children have been expected to play a significant role (e.g. handwashing, social distancing) in reducing the transmission of the virus.

This study resulted from an international collaboration between six countries; UK, Australia, Sweden, Brazil, Spain and Canada. Each of these countries experience of COVID-19 has been different. There was a notable difference in the variation in death rates with relatively few deaths in Australia but much a higher rate in the UK and Brazil. The authorities in the different countries asked their populations to adhere to different approaches to mitigate the transmission of the virus (Table 1), different approaches to how key health messages and information were shared with children about COVID-19 (Table 1), and different terms were used by children, parents and the media in relation to COVID-19 e.g. coronavirus (UK, Australia) and corona (Sweden). Some countries such as Canada held national press briefings specifically for children and developed LEGO® animations to share health messages from the Prime Minister with children. In other countries, such as England, the children’s minister answered questions posted by children. Information for children such as colourful books and videos have been generated and shared through social media, the internet and school (Table 1). It is not known if this information reaches its intended audience or what other sources of information about coronavirus children access either independently or through their parents, friends or communities. We know that children’s health literacy is heavily influenced by family and organisational systems [9], and we were keen to explore aspects of children’s health literacy (access to information, information exchange, information preferences, perceived understanding) during a pandemic across a range of countries and contexts.

**Table 1:** Examples of the different approaches to mitigate transmission of COVID-19 and provide information to children about COVID-19 (coronavirus) within the participating countries during the time of the study.

Country	Different approaches to mitigate transmission of COVID-19	Examples of different forms/channels of information
United Kingdom	Lockdown was introduced on the 23^rd^ of March and people were asked to ‘stay at home to save lives’. Children were schooled at home, apart from the children of key workers. Shops other than those selling essential provisions were closed, as were parks, playgrounds and leisure facilities. Group activities and non-essential travel was stopped.	Information for children such as colourful books and videos have been generated by individuals, charities and health organisations and shared through social media, the internet and school. The children’s minister answered questions posted by children on several occasions.
Sweden	Requirements for social distancing was minimized for children in Sweden. Children aged 6–16 years attended school during the whole period of pandemic. Older children (aged 16–19 years) and many adults were required to work remotely. Group activities of more than fifty people, e.g. leisure and club activities were cancelled. Many shops were closed, people were only allowed to travel 1–2 hours from home and children were not allowed to visit their grandparents	The Public Health Agency distributed information about COVD-19 through posters, TV, radio and internet. There is information on the agency´s website adapted for children (information about the virus and how it is spread, and how to stay safe) including a YouTube video. Children received information in school via national education programme.
Australia	Australian borders were closed to all non-residents on 20^th^ of March. States started closing borders in mid- late March with each one declaring a public health emergency. Social distancing rules were imposed on 21^st^ March and state governments started to close non-essential services, for children this meant closure of playgrounds, parks, pools and libraries. Schools closed in early April and, apart from children of essential workers, children ‘learnt from home’. Individuals were asked to ‘stay home to save lives’ and ‘flatten the curve’	Children’s television programs aired specific episodes on COVID-19 for example Play School (preschool—age 6) and Behind the News (age 8–13). Dedicated health and government news conferences answered questions from children. Schools communicated via fact sheets, posters, social media, emails and SMS to update parents. Information for children was often directed to parents in the first instance e.g. ’how do I talk to my child’. Radio and parenting websites also gave practical guidance to parents e.g. stopping the spread and physical distancing.
Canada	Lockdown was introduced differently (schedule and directives) for each province. Generally, public gathering restrictions started on the 12th of March, schools closed the following week and all non-essential businesses closed on the 23rd of March. Travel restrictions were put in place between many provinces and Territories. Throughout Canada, most children were home-schooled during the confinement with wide variations of teacher involvement.	Some daily briefings given by the Prime minister of Canada included a few words directly to the children. Quebec’s Prime minister also had a few briefings targeting children. Influencers on social media were asked to reinforce the public health guidance to young people. Online resources were available on the Government of Canada website.
Brazil	Social distancing was implemented autonomously by each of the states within Brazil. The state of São Paulo, started lockdown in the second and third week of March with the closure of schools and universities. Some states tried to enforce lockdown, but in some cases without much success. Brazil is experiencing one of the highest global death rates/million population from COVID-19.	Specific content for children (animation, videos and comic book) was provided by schools, by the ministry of health, pediatric societies, universities, and hospitals.
Spain	Lockdown started on the 13^th^ of March for everybody in the country. Initially for 2 weeks but at the end this was extended until the 20^th^ of June. Children were required to stay indoors for six weeks.	There were no official messages from the government addressed to children or young people. There were spots on TV from the Ministry of Health to increase the awareness about the use of masks and social distancing requirements, using cartoons, but these were addressed to all ages of the population. Specific institutions such as the children’s hospitals have designed specific content for parents and children.

This international study aimed to examine aspects of children’s health literacy; information sources they were accessing, their information preferences, their perceived understanding of and their reported information needs in relation to COVID-19. The study also explored the role parents/caregivers play in aspects of their child’s health literacy and factors that influence their decisions to share, discuss, direct or limit their child’s access to information. This study was conducted relatively early in the trajectory of the pandemic, whilst many countries were requiring children and families to mitigate the transmission of the virus by staying at home. It is important to understand children’s access to and preferences for information in order to ensure that optimal strategies are developed to communicate information within this and any future pandemics.

## Materials and methods

This study used a prospective online survey using the Survey Monkey platform to collect quantitative and qualitative information from children and parents/caregivers.

The survey was developed and pre-tested with nine children (aged 9–16 years) and five parents from the UK through remote consultation (email, telephone). The children and parents were either known to the researchers or members of The Forum (a young peoples’ consultations and engagement group). The consultation resulted in amendments being made to the content, format, language and appearance of the survey and recruitment materials.

The children’s survey was designed to foreground their abilities and skills, focussing on short closed and open text questions and a word association question (Table 2). The survey also asked children to draw and label a picture about COVID-19/coronavirus/corona and ‘why we are being asked to stay at home’ (this element of the study will be reported elsewhere). The survey for children focussed on; their access to information about COVID-19 and preferences for receiving information, their knowledge about the virus, their information needs and their thoughts associated with the pandemic (Table 2). The survey was targeted at 7–12 year olds, reflecting middle childhood and a critical age for developing health literacy skills [10] as this is when they are starting to shape their own constructs of healthy actions [11]. The survey for parents/caregivers included five closed questions and five questions seeking longer text responses. The parent/caregiver survey was structured around; their access to information about COVID-19 (sources, frequency of accessing sources), their child’s access to information sources and whether and how they chose to share/not share information about COVID-19 with their child (Table 2). The surveys of children and parents were not linked, both could participate independently of one another.

**Table 2:** Questions in the child and parent/caregiver survey.

**Children’s survey**	
Where have you got information from about the coronavirus?	Multiple choice question with ‘other option’ (mum/dad/carer, friends, TV, Children’s TV programme, Internet, Social media, School, You Tube, Radio, Newspapers/magazines, NHS websites, I haven’t looked at any information, government information/press releases)
From the choices above, which is the main way you get your information about the coronavirus?	Open text
Generally, has the information been easy to understand	Multiple choice (very easy to understand, easy to understand, difficult to understand, very difficult to understand, not sure)
How would you like to get information about the coronavirus?	Open text
Please tell us three things you know about coronavirus	Open text
Please tell us about three things you would like to know about coronavirus	Open text
How much do you think you know about coronavirus?	Multiple choice (I know lots and lots, I know quite a bit, I know a little bit, I don’t know much)
I find talking about the coronavirus…	Multiple choice (interesting worrying, fine, boring, useful, I don’t talk about it, other)
Please tell us 3 words you think of when thinking about the coronavirus	Open text
Could you draw and label a picture to explain ‘why we are all trying to stay at home during the coronavirus?	Open
**Parent/caregivers’ survey questions**	
Where do you access information about coronavirus (COVID-19)	Multiple choice (Family, friends, TV, Leaflets, Internet news, WhatsApp, NHS websites, health charities, Children’s TV channels, radio, social media, newspapers and magazines, I try and avoid any information, other)
From the choices above where is the main place you get information about coronavirus?	Open text
How often do you access information about COVID-19?	Multiple choice (multiple times a day, once day, every other day, every 3–4 days, once a week, other)
How much do you feel you know about coronavirus?	Likert scale (I know enough about COVID-19, I don’t know enough about COVID 19, I am not sure)
Where does your child get their information about coronavirus?	Tick as many as applicable (parent/carer, friends, You tube, social media, internet, TV, Newspapers/magazines, school, radio, they have not accessed any information, I restrict their access to information, other)
How do you decide what information to share with your child about coronavirus (COVID-19)?	Open text
Is there any information you choose not to share with your child about coronavirus (COVID-19)?	Open text
I find talking about coronavirus with my child	Multiple choice (makes them less worried, makes them more worried, I am unsure how this makes my child feel, I do not talk to my child about coronavirus)
What kind of information about coronavirus (COVID-19) do you think would be helpful to your child?	Open text
How do you think your child would like to receive information about coronavirus (COVID-19)?	Open text
Please add below any other thoughts you have about how children access information about coronavirus?	Open text

Three broad demographic questions (age, area and country) were collected from both children and parents/caregivers; responses were anonymous and did not identify the participant. We asked both children and parents if they/their child were attending school or being home-schooled and if who was living in the family home had changed as a result of COVID-19. The survey was translated into Spanish, Portuguese, French Canadian and Swedish and there was some minor tailoring of some questions/response options to suit the context of individual countries, including the term used to describe COVID-19.

The survey opened in the UK (9^th^ of April 2020), Australia (20^th^ April 2020), Canada (27^th^ April 2020), Brazil (29^th^ April 2020), Spain (3^rd^ May 2020) and Sweden (22^nd^ May 2020). Data collection closed within all countries on the 1^st^ of June 2020.

### Recruitment

Children aged 7–12 years of age and parents/caregivers in the collaborating countries were eligible to participate in the study. To reach as many families as possible we used professional and personal networks and social media (Twitter, Facebook) platforms. A short information flyer about the study was included in each post/communication to accompany the survey links. More than one child and one parent from a household could participate and children and parents could participate independently from each other.

### Analysis

Numerical data were analysed using SPSS. The study is descriptive and therefore inferential statistics were not conducted, descriptive statistics (percentages and frequencies) have been used to examine the data.

The open text data were analysed using qualitative content analysis [12]. The initial analysis was led by the team in the UK, who inductively constructed a coding framework. We met remotely to discuss and explore interpretations and the developing codes and themes. The framework further developed through additions and reorganisations when data from other countries were added and integrated.

### Ethical approval

The study was approved by ethical review boards within the employing organisation of lead academics within each participating country; UK (Edge Hill University Faculty of Health Social Care and Medicine Research Ethics Committee CYPF 23), Australia (Tasmanian Human Research Ethics Committee 21702), Brazil (Botucatu Medical School Research Ethics Committee opinion n° 3.994.298), Spain (approval was not deemed necessary by the ethics committee), Canada (Comité d’éthique de la recherche (CER) Certificate number 2021–1163, UQO 2010–1163) and Sweden (Swedish Ethical Review Authority, DNR2020-02351). Information at the beginning of the survey provided a brief explanation of what participation in the study entailed and outlined that by submitting the survey, a parent/caregiver (consented) or a child agreed (assented) to take part in the project. The research ethics committees waived the need for written parental consent or written assent for participation. Information at the end of the survey directed children and parents to websites with resources to support them (of relevance to each participating country).

## Findings

A total of 390 children and 1,230 parents/caregivers participated in the study distributed across the 6 participating countries (Table 3). The average age of the children who completed the survey was 9 years (SD 1.7).

**Table 3:** Basic demographic information of the participants.

	UK	Sweden	Australia	Canada	Brazil	Spain	Total
**Parent/caregiver participants**	**279 N (%)**	**235 N (%)**	**123 N (%)**	**80 N (%)**	**132 N (%)**	**381 N (%)**	**1230 N (%)**
**Role**							
Mother	249 (89%)	205 (87%)	110 (89%)	74 (93%)	116 (88%)	295 (77%)	1049 (85%)
Father	23 (8)	25 (11%)	11 (9%)	6 (8%)	14 (11%)	86 (23%)	165 (13%)
Caregiver	5 (2%)	4 (2%)	0	0	0	0	10, (0.01%)
Missing	2 (1%)	0	2 (2%)	0	2 (2%)	0	6 (0.5%)
**Child Participants**	**156**	**50**	**49**	**25**	**58**	**52**	**390**
	**N (%)**	**N (%)**	**N (%)**	**N (%)**	**N (%)**	**N (%)**	**N (%)**
**Age (M, SD)**	9.24, 1.7	9.69, 1.8	9.3, 1.8	9.5, 1.6	9.1, 1.7	9.3,1.6	9.3, 1.7
Missing	1	1	2	1	0	0	
**Self-reported school attendance** (N, %)							
Accessing school work at home	147 (94%)	3 (6%)	31 (63%)	23 (92%)	58 (100%)	51 (98%)	313 (80%)
Going into school	-	46 (92%)	4 (8%)	1 (4%)	0	0	51 (13%)
Mix of school work at home and school	7 (5%)	0	12 (25%)	0	0	0	19 (5%)
Missing	2 (1%)	1 (2%)	2 (4%)	1 (4%)	0	1 (2%)	7 (2%)

The findings are structured in two sections; the first section highlights how information relating to COVID-19 was accessed by children and parents/caregivers, including how parents chose to facilitate or limit their child’s access to information. The second section relates to children and parent/caregivers’ reported understandings of COVID-19. Despite there being different approaches to mitigating the spread of the virus within the participating countries and different rates of COVID-19 related deaths, there were commonalities in the responses of the children and the parents.

### Accessing information about COVID-19

There were similarities in the sources of information children accessed across the participating countries. Most children accessed information about COVID-19 through their parents or caregivers (89%, n = 347) (Table 4), with the exception of children in Sweden who reported their main source of information as from school (90%, n = 45). This is of note as children participating in this study in Sweden were still attending school in person, whereas children in the other participating countries were mainly learning at home. Other commonly reported sources were school and the TV, although many children reported that these were child-orientated channels and programmes ‘*on a specific channel because I think just seeing the news on TV is harmful*’.

**Table 4:** Children’s self-report of their access to information about COVID-19.

	UK	Sweden	Australia	Canada	Brazil	Spain
Child participants	156	50	49	25	58	52
**Children’s self-report of where they access information about COVID-19 (response to multiple choice options)**						
1^st^ highest response	Parent/caregivers 135 (87%)	School 45 (90%)	Parent/caregiver 48 (98%)	Parent/caregiver 23 (92%)	Parent/caregiver 49 (85%)	Parent/caregiver 48 (92%)
2^nd^ highest response	School 105 (67%)	Parent/caregiver 44 (88%)	TV 34 (69%)	School 11 (44%)	TV 41 (71%)	TV 41 (79%)
			School 34 (69%)			
3^rd^ highest response	TV 91 (58%)	Children’s TV News 39 (78%)	Friends 20 (41%)	TV 9 (36%)	School 32 (55%)	School 26 (50%)
				Prime minister conference 9 (36%)		
**Children’s self-report of where they would like to access information about COVID-19 (open text response, three most frequent responses)**						
1^st^ highest response	Parent/caregiver 54 (35%)	Teachers/school 18 (36%)	Parent/caregiver 15 (31%)	Parent/caregiver 8 (32%)	Animations 10 (17%)	Parent/caregiver 15 (28%)
2^nd^ highest response	TV and TV news 38 (24%)	TV and TV news 12 (24%)	TV and TV news 12 (%)	TV and TV news 4 (16%)	Websites for kids/You tube 8 (14%)	TV and TV news 15 (28%)
3^rd^ highest response	Teachers/school 14 (9%)	Parent/caregiver 7 (14%)	Websites for kids/YouTube 6 (12%)	Animation 2 (8%)	TV and TV news 7 (12%)	Websites for kids/You Tube 7 (13%)
		I do not know 7 (14%)				

The sources children reported accessing for information (parents/caregivers or school) about COVID-19 generally matched their preference for where they received or accessed information (parents/caregivers or school) about COVID-19. The exception to this was Brazil where children reported parents/caregivers as their main source of information (85%, n = 49), but they would like to receive information via animations (17%, n = 10). However, across the participating countries, children’s preferences for information did not match those reported by parents/caregivers. Despite parents/caregivers recognising themselves as the primary source of information for their child (93%, n = 1,147), they reported that their child would prefer to receive information via animations, TV, games/quizzes and adults in a position of trust e.g. doctors/scientists (Table 5). Children reported their preference was to receive information through their parents.

**Table 5:** Parents/caregivers’ self-report of where they and their child access information about COVID-19.

	UK	Sweden	Australia	Canada	Brazil	Spain
Parent/caregiver participants	279 N (%)	235 N (%)	123 N (%)	80 N (%)	132 N (%)	381 N (%)
**Parent/caregivers’ self-report of where** **they** **access information about COVID-19 (multiple choice response, three most frequent responses)**						
1^st^ highest response	TV 209 (75%)	TV 168 (71%)	Government websites 92 (75%)	Premiers conference 61 (76%)	Government websites 73 (55%)	TV 290 (76%)
2^nd^ highest response	Internet news 158 (57%)	Public health website 156 (66%)	Social media 82 (67%)	Social media 55 (69%)	TV 72 (55%)	Newspapers/magazines 174 (46%)
3^rd^ highest response	Public health website (NHS) 140 (50%)	Internet 144 (61%)	TV 68 (55%)	TV 51 (64%)	Internet 66 (50%)	Internet 173 (45%)
	Social media 140 (50%)					
**Parent/caregivers’ report of where their child accesses information about COVID-19 (Multiple choice response, three most frequent responses)**						
1^st^ highest response	Parent/carer 260 (93%)	Parent/carer 228 (97%)	Parent/carer 119 (97%)	Parent/carer 6 (95%)	Parent/carer 118 (89%)	Parent/carer 343 (90%)
2^nd^ highest response	TV 122 (44%)	School 206 (88%)	School 64 (52%)	TV 30 (38%)	TV 57 (43%)	TV 188 (49%)
3^rd^ highest response	School 71 (25%)	TV 101 (43%)	TV 46 (37%)	Social media 19 (24%)	School 40 (29%)	School 112 (29%)
**Parent/caregivers’ report of where** **their child** **would like to access information about COVID-19 (open text response and three most frequent responses for each participating country)**						
1^st^ highest response	Animations 128 (46%)	Animations 98 (42%)	Animations 69 (56%)	TV/child friendly websites/news for children 22 (28%)	Animations 79 (60%)	Talks by a trusted adults they do not know (scientists/doctors/teachers) 131 (34%)
2^nd^ highest response	Games/quiz 57 (20%)	Talks by a trusted adults they do not know (scientists/doctor) 50 (21%)	Games/quiz 29 (24%)	Animations 18 (23%)	Games/quiz 53 (40%)	Animations 124 (33%)
3^rd^ highest response	Talks by a trusted adult they do not know (scientists/doctors) 42 (15%)	Games/quiz30 (13%)	Talks by a trusted adult they do not know (scientists/doctors) 27 (22%)	Games/quiz 15 (19%)	Talks by a trusted adult they do not know (scientists/doctors/teach ers) 8 (6%)	Games/quiz 87 (23%)

The role of parents/caregivers in facilitating or limiting their child’s access to information about COVID-19 was clearly demonstrated in the findings. Many of the parents/caregivers in this study made choices to shield (13%, n = 160) or filter/limit (37%, n = 451) their child from information about COVID-19 (Table 6). Some parents explained that their decision to ‘*shield my kids from the worst of it’* was driven by a desire to protect them from the most distressing pieces of information and news, to prevent them *‘worrying too much’* or ‘*being too scared*’. In contrast some parents/caregivers (20%, n = 242) reported the importance of their child ‘*knowing everything about it’* and not having information ‘*hidden from them*’ and ‘*feeling like adults are lying to them and holding stuff back*’. Many of the responses identified that it was important to acknowledge individual information needs and preferences for gaining information about COVID-19, ‘*I share information which is appropriate for her in a way which makes sense to her*’. This is important as in many cases, parents/caregivers were the main source of information for children whom were in ‘lockdown’ and their contact with people outside their homes was limited. Some of the responses from parents in Sweden indicated that their children still attending school impacted on their access to information; they trusted school to provide information to their child or did not *‘have any power over the information they get*. *They are in school and hear lots of information*”.

**Table 6:** Parents/caregivers’ decisions, choices and approaches to their child’s access to information about COVID-19.

	Country	Frequency of responses N (%)	Representational quote selected from each country
**Parent/caregivers’ report of how they share information about COVID-19 with their child (open text responses, content analysis and reporting of the most frequent responses)**			
Shielding my child from information	UK (n = 279)	**69 (25%)**	I try to shield my kids from the grim reality
	Sweden (n = 235)	**8 (3%)**	Trying not to watch all the news while the kids are present.
	Australia (n = 123)	**9 (7%)**	The bare minimum they need to know
	Canada (n = 80)	**6 (8%)**	So as not to scare him, I keep information from him.
	Brazil (n = 132)	**13 (10%)**	The only accessible way to know about coronavirus is on TV, therefore we select what we watch in front of them
	Spain (n = 381)	**56 (15%)**	We don’t talk about it
Diluting, filtering and adapting the information shared with my child	UK (n = 279)	**95 (34%)**	We have kept it very simple, just sharing what is necessary to keep them safe and well
	Sweden (n = 235)	**71 (30%)**	I tell the most important things but save the scariest details
	Australia (n = 123)	**63 (51%)**	What is relevant, age appropriate, in simple language and nothing too overwhelming
	Canada (n = 80)	**38 (47%)**	Information that will help to keep them safe ie social distancing, extra hand washing, sanitizing and explain to them why we are doing home learning and why things are closed.
	Brazil (n = 132)	**49 (24%)**	I share information about prevention, hygiene. But I don’t share information about the death rate or sad news
	Spain (n = 381)	**135 (35%)**	I share the minimum. Only its existence, consequences such as not being able to go to school, not being able to leave and the message that all this will be fixed.
Giving information by responding to my child’s questions	UK (n = 279)	**67 (24%)**	We are answering his questions when he asks but not offering information he hasn’t asked for.
	Sweden (n = 235)	**48 (20%)**	In the first place I let my child raise it himself, then we talk about it and I answer questions if they have any
	Australia (n = 123)	**21 (7%)**	I ask my daughter if she has any questions and I answer them in a way appropriate to her, so that she understands and can properly comprehend the information.
	Canada (n = 80)	**13 (16%)**	I answer their questions. I don’t supply too much info unless they have questions.
	Brazil (n = 132)	**10 (8%)**	I try to answer only what he asks me
	Spain (n = 381)	**34 (9%)**	I respond to all my child’s questions and we discuss together any doubts or further comments that she may have.
Carefully sourcing accurate information to share with them	UK (n = 279)	**10 (4%)**	I share anything that I believe to be fact. No sensationalism. I only share when they ask or if there is a significant piece of news
	Sweden (n = 235)	**16 (7%)**	We discuss and agree on what is reasonable to share.
	Australia (n = 123)	**33 (27%)**	We ensure it is factual and age appropriate
	Canada (n = 80)	**18 (23%)**	I ensure all is ‘fact’ and science based {real news-data}, not fake news nor ’dumbing down’ of content.
	Brazil (n = 132)	**19 (14%)**	We find and then provide information using appropriate language
	Spain (n = 381)	**107 (28%)**	I look up serious information which is not sensationalist
Sharing information with my child in an honest and open way	UK (n = 279)	**33 (12%)**	I don’t hide anything about the coronavirus with my children, but I use age appropriate language when explaining the more distressing aspects.
	Sweden (n = 235)	**32 (14%)**	He gets to know what he wants via news. We talk about to see what image he has, what he thinks and whether he is worried or not
	Australia (n = 123)	**33 (27%)**	I am just open and honest about it all. We discuss openly
	Canada (n = 80)	**18 (23%)**	We have open, honest conversations so that my child can understand what this pandemic is about, how it spreads and how to stay safe.
	Brazil (n = 132)	**19 (14%)**	I decided that he needs to know everything about the subject because it is very important to know how to take care and also see that it is very serious.
	Spain (n = 381)	**107 (28%)**	No restriction, just do not overload them with information and adapt it to their age
Children being autonomous in addressing their information needs	UK (n = 279)	**5 (2%)**	We think it is important to be led by what they want to know.
	Sweden (n = 235)	**22 (9%)**	We have come to an agreement with the children that we should not talk too much about corona at home
	Australia (n = 123)	**0**	-
	Canada (n = 80)	**5 (6%)**	She’s old enough to decide for herself.
	Brazil (n = 132)	**0**	-
	Spain (n = 381)	**0**	-
**Parent/caregivers’ report of information they choose** **not** **to share with their child (open text responses, content analysis and reporting of the most frequent responses)**			
We don’t talk about the death rates	UK (n = 279)	**132 (48%)**	We have said some people have died from it but not to worry
	Sweden (n = 235)	**42 (18%)**	We try to avoid death figures and things that can be perceived as scary
	Australia (n = 123)	**63 (51%)**	We don’t talk about death, they know people have died but we don’t go into details.
	Canada (n = 80)	**36 (45%)**	I will turn the radio off if they are talking about death counts.
	Brazil (n = 132)	**52 (40%)**	I don’t like him to see the deaths shown on TV everyday
	Spain (n = 381)	**105 (28%)**	We don’t talk about the number of deaths
We filter/adapt the information	UK (n = 279)	**7 (3%)**	We try to be as open with him as much as possible but he is still young so we don’t want him to be frightened. We explain that people with the virus may die but we don’t give him detailed information such as the number of people that have died.
	Sweden (n = 235)	**26 (11%)**	What feels important to me and useful to the child
	Australia (n = 123)	**6 (5%)**	We don’t share information about what is happening outside of Australia
	Canada (n = 80)	**2 (3%)**	We don’t hide anything but we don’t explain everything, we keep facts that could cause anxiety to us.
	Brazil (n = 132)	**8 (6%)**	We do not share political developments and fake news—information that do not add value
	Spain (n = 381)	**42 (11%)**	I have diluted the information to allow them to understand the danger without scaring them.
We do not withhold information	UK (n = 279)	**58 (21%)**	Not really. Death is a reality, and death from disease is too. It’s important to be open but calm.
	Sweden (n = 235)	**8 (3%)**	We share as much current information as possible at the level my child understands
	Australia (n = 123)	**19 (15%)**	I’m not too concerned about them knowing about it
	Canada (n = 80)	**33 (41%)**	I tell them everything in an age appropriate language and terms. I don’t try to make up answers to questions where I don’t know or where the evidence is uncertain.
	Brazil (n = 132)	**75 (57%)**	No. I think it is essential that he knows all the implications and why these measures have been taken are important
	Spain (n = 381)	**167 (44%)**	We have no restrictions on what we share
We do not share the risks of how children or people we know could get poorly/die	UK (n = 279)	**16 (7%)**	We don’t mention that it could affect their grandparents
	Sweden (n = 235)	**24 (10%)**	Trying to keep away from how dangerous it can be to our loved ones
	Australia (n = 123)	**9 (7%)**	We also don’t talk about children dying or other adults close in age to us
	Canada (n = 80)	**12 (15%)**	Victims that are children
	Brazil (n = 132)	**2 (2%)**	About negative repercussions, especially in children.
	Spain (n = 381)	**11 (3%)**	We don’t share traumatic information or risks to children

The decisions of parents/caregivers to shield or limit their child’s access to information in order to reduce feelings of anxiety about COVID-19 was interesting, as most parents/caregivers across all countries reported that talking about COVID-19 with their child made their child feel less worried (n = 801, 65%), as opposed to more worried (n = 169, 14%).

### Understanding about coronavirus (COVID-19)

Children and parent/caregivers were asked to select on a multiple choice question how much they felt they knew about COVID-19. Both children (Table 7) and parents (Table 8) reported that they had good levels of knowledge about COVID-19.

**Table 7:** Children’s self-report of how much they know about COVID-19.

	UK	Sweden	Australia	Canada	Brazil	Spain	Total
Child participants	156 N (%)	50 N (%)	49 N (%)	25 N (%)	58 N (%)	52 N (%)	
I know lots and lots	26 (17%)	3 (6%)	3 (6%)	1 (4%)	8 (14%)	1 (2%)	42 (11%)
I know quite a bit	76 (49%)	30 (60%)	23 (47%)	11 (44%)	46 (80%)	27 (52%)	213 (55%)
I know a little bit	41 (26%)	7 (14%)	17 (35%)	11 (44%)	2 (3%)	19 (37%)	97 (25%)
I don’t know much	12 (8%)	6 (12%)	5 (10%)	2 (8%)	2 (3%)	5 (10%)	32 (8%)
Missing	1, 0.6	4 (8%)	1, 2	-	-	-	6 (2%)

**Table 8:** Parent/caregivers’ self-report of how much they know about COVID-19.

Parent/caregiver participants	UK N (%)	Sweden N (%)	Australia N (%)	Canada N (%)	Brazil N (%)	Spain N (%)	Total
I know enough about COVID-19	193 (69%)	171 (73%)	104 (85%)	55 (69%)	76 (58%)	178 (47%)	781 (63%)
I don’t know enough about COVID-19	48 (17%)	24 (10%)	7 (6%)	10 (13%)	30 (23%)	101 (27%)	220 (18%)
Not sure	38 (14%)	39 (17%)	12 (10%)	15 (19%)	26 (20%)	101 (27%)	232 (19%)
Missing	0	0	0	0	0	1 (0.5%)	1 (0.1%)

Children were asked to report three things they knew about coronavirus in an open text format. There were 115 different items identified by the 390 children who participated; most responses were short. The three most frequent items identified by children in each country are noted in Table 9. The findings show that children were aware that coronavirus ‘*spreads really quickly’ (*28%, n = 110*)*, that ‘*many people around the world are infected or have died*’, it ‘*started in China*’ (22%, n = 87) and ‘*is dangerous’* (13%, n = 51). Other frequently identified pieces of information included that ‘*we need to stay home to save lives*’ (n = 54, 14%), ‘*it can kill you*’ (n = 30, 8%) and ‘*it is a stupid virus*’ (n = 30, 8%). There were similarities noted between children’s responses from the different participating countries, despite there being differences in the contexts within each country (infection and death rates, social distancing rules). Some children wrote longer quotes which demonstrated feelings about information being hidden from them *‘people play it down and tell me it can’t kill people*, *but people are dying each day***’.**

**Table 9:** Children’s self-report of their knowledge and information needs relating to coronavirus.

	UK	Sweden	Australia	Canada	Brazil	Spain
Child participants	156 N (%)	50 N (%)	49 N (%)	25 N (%)	58 N (%)	52 N (%)
**Children’s self-report of what they know about coronavirus (COVID-19) (response to open text response, the three most frequent responses for each country)**						
1^st^ highest response	It is killing people/it is deadly 45 (29%)	It is mostly dangerous for old people 12 (24%)	It spreads easily/is infectious/contagious 18 (39%)	It spreads easily/is infectious/is contagious 11 (44%)	It is dangerous/bad 17 (29%)	It spreads easily/is infectious/is contagious 26 (50%)
2^nd^ highest response	It spreads easily/is infectious/contagious 43 (28%)	It started in China 10 (20%)	It started in China 13 (28%)	It is killing people/it is deadly 5 (20%)	It spreads easily/is infectious/contagious 12 (21%)	It started in China 26 (50%)
3^rd^ highest response	It started in China 38 (24%)	It is dangerous/bad 8 (16%)	It is killing people/it is deadly 11 (24%)	We need to stay home (to save lives) 5 (20%)	It is killing people/it is deadly 11 (19%)	It is mostly dangerous for old people 9 (17%)
				It is mostly dangerous for old people 5 (20%)		It is killing people/it is deadly 9 (17%)
**Children’s self-report of what they want to know (open text response, three most frequent responses from each country)**						
1^st^ highest response	When will it stop/end/go away 37 (24%)	When will it stop/go away? 20 (40%)	When will it stop/end/go away 16 (35%)	When will it stop/end/go away? 6 (24%)	Can we cure it? 22 (38%)	Can we cure it? 16 (%)
2^nd^ highest response	How did it start/where did it come from? 34 (22%)	How did it start/where did it come from? 16 (32%)	How did it start/where did it come from? 15 (33%)	When will a vaccine be available? 5 (20%)	When will a vaccine be available? 19 (33%)	When will it stop/end/go away? 14 (27%)
3^rd^ highest response	When will a vaccine be available? 15 (10%)	When will a vaccine be available? 5 (10%)	When will a vaccine be available? 3 (7%)	How did it start/where did it come from? 4 (16%)	When will it stop/end/go away 18 (31%)	How did it start/where did it come from? 12 (23%)
		Can we cure it? 5 (10%)	Can we cure it? 3 (7%)			

The most frequently reported information needs from children were linked to ‘*when it will go away’* (28%, n = 111), ‘*how and where did it start*’ (21%, n = 81) and *‘when and whether a cure or vaccine will be found (23%*, *n = 88)’*. Other frequently identified information needs included *‘how does it make you poorly’*, *‘when can we go back to school’* and *‘what does the coronavirus actually look like’*. Some children expressed that they did not want any more information about coronavirus, ‘*it is boring*’, ‘*I am sick of hearing about it*’ or ‘*I don’t want to know about it*, *because it’s killing people and that makes me sad*’. In total 139 different questions were identified from the 390 children who participated in the study. These questions were similar across the participating countries, despite the different contexts the children were living in.

## Discussion

The focus of health literacy initiatives in relation to children and COVID-19 has been to develop accessible information through ensuring materials are accessible, appealing, readable and digestible [13]. These initiatives, whilst worthy and useful, fail to acknowledge the importance of the wider aspects of health literacy and how family and societal expectations and assumptions can limit children’s access to meaningful information during a pandemic. This study shows that despite accessible child-friendly information being available, wider aspects of health literacy such as familial (parents filtering and shielding children from information) and societal (understandings about children’s rights and vulnerabilities) challenged children’s ability to access and understand some aspects of the response to COVID-19. This paper argues that the current approach to sharing COVID-19 information with children in many countries has been framed according to adult perspectives and foregrounds concerns around children’s vulnerabilities and lack of competence.

Children in this study demonstrated that although they had information and self-reported understanding of some key elements around the COVID-19 pandemic, they sought answers to important questions. Despite the many child-centred resources written and developed to address children’s information needs, children across most of the participating countries were heavily dependent on their parents/caregivers for information about COVID-19 during the pandemic. The exception to this being children in Sweden who continued to attend school and gain most of their information via school. Parents’ role as the lynchpin to their child’s access to COVID-19 information, reflects existing evidence that parents are often the primary source of children’s health information [10]. This role is important as although some parents reported adopting an open and honest approach to sharing information, some parents chose to shield their child from some of the upsetting news about COVID-19, and many diluted, filtered or adapted the information they shared with their child. As noted within other studies, from other contexts, parents/caregivers often filter or limit their child’s access to health information [14] in order to protect them from upsetting news [15] and offer reassurance in place of facts [14]. Despite parents’ choices, many of the children in this study reported knowing that people were dying every day from the virus and that the virus was dangerous and spreading quickly.

Although it is known that children’s exposure to distressing imagery and news can cause them to experience anxiety [16], shielding children completely from information leaves them to ‘fill in the blanks’ on their own [17], using their imagination and pieced together information. Our study supports previous work which shows how communication with parents and family members is an important element for children’s health literacy [18, 19]. The interaction within a family can help children construct and develop their understanding [20] of a topic through layering their knowledge and apply meaning [19]. Therefore, listening to what children believe about COVID-19 is essential. Providing children with an accurate explanation that is meaningful to them will ensure that they do not feel unnecessarily frightened [17]. The need for clear targeted messaging for children at a community and societal level acknowledges that parents may not always choose to share, know the information themselves or know how to discuss difficult topics with their child.

Within many Western societies, children are perceived as vulnerable and innocent and are therefore often only allowed access to certain types of knowledge; their access to information is censored [21]. Although health education initiatives should embody children’s right to be ‘heard and listened to’ as acknowledged by the UNCRC [22], evidence has shown how such initiatives can often be framed by adult agendas and concerns [19] and not acknowledge children as social actors [23]. There has been a call for children to be framed as ‘equals’ and ‘co-learners’ in how knowledge is created [24]. Certainly within the current pandemic there are uncertainties at all levels but there are opportunities for children to learn alongside parents and communities (schools, local organisations). Within some of the participating countries (notably Canada and Sweden), the approach to sharing information seemed to acknowledge children as citizens and health messages were addressed at a national level and in an equitable way to those of adults. Whereas in other countries (Brazil), political disquiet may have influenced how information was created and shared with children. Our paper supports the understanding of health literacy proposed by Nutbeam [25] as an ‘interaction between the person and their environment’ and recognises the importance of context on an individuals’ health literacy, in this case children’s, ability to obtain, understand and act on health information.

**Fig 1:**
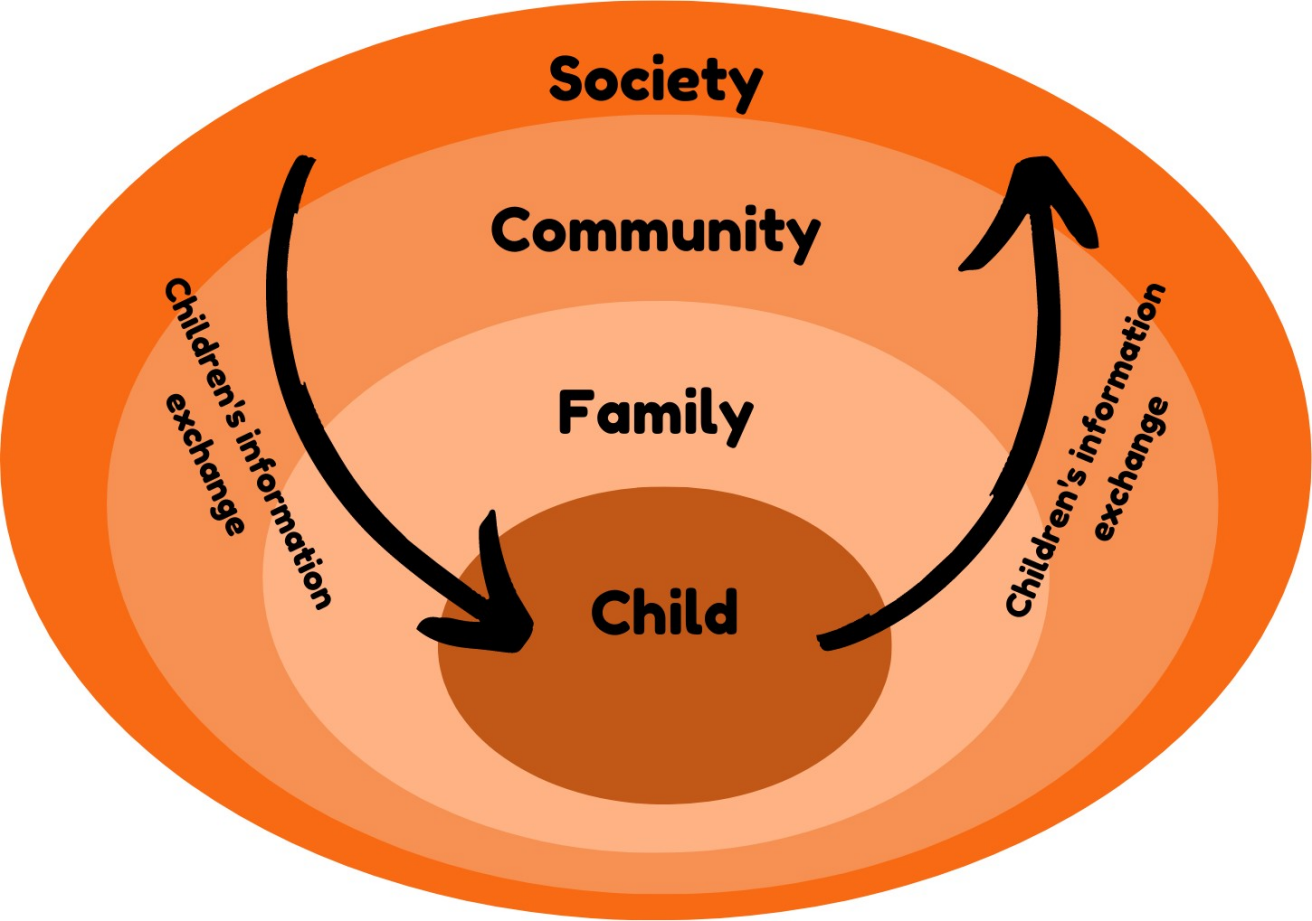
Children’s information exchange in relation to COVID-19.

There is a need for a multi-source approach to how children receive information during a pandemic. We propose that children need access to reliable and meaningful information from a range of sources (individual, family community/school and society) during the pandemic in order to make sense of the world and should be acknowledged as active participants in the management of the pandemic (Fig 1).

Children in our study were able to identify their information needs. We argue they should be empowered at a familial, community and societal level to raise and discuss these information needs and concerns. Our study supports previous work that children’s health literacy should not be considered as an individual characteristic or as ‘individual agency’ [26] but embedded within family, community (school, local community organisations etc) and wider societal health literacy practices [27].

This study contributes new insight into children’s access to and understanding of information about COVID-19 and highlights the importance of families, communities and society on these aspects of children’s health literacy. Further research should examine how access to credible information and levels of understanding influence children’s actions and decisions during the pandemic.

### Limitations

The sample for this study was a convenience sample of those children and parents/caregivers who self-selected to participate, and the numbers of responses are low compared to the number of children who live within each country, therefore there are limitations in the representativeness of the findings. The survey was intentionally designed to be short to encourage completion; however, as a result demographic data including gender, socio-economic background, presence of special educational need or disability or parental employment were not collected. Absence of these data limits our understanding of how these factors may have influenced perceived knowledge or communication patterns within a family. Children may have completed the survey in the presence of their parents and therefore felt the need to respond in a way desirable to their parents. We did not obtain an objective measure of knowledge levels, instead children and their parent/caregivers’ were asked for a self-report of their perceived knowledge level. The survey was developed specifically for this study, and although we consulted children during the development phase, there may have been items which were not clear to children. The survey was conducted in a rapidly changing landscape and although data were collected at the same time across the participating countries, the spread of the virus evolved at a different pace across the countries. There were different approaches within the participating countries to how the spread of the virus was mitigated (Table 1), the majority of children in the study, apart from those in Sweden, were at home and their access to sources of information independent to their parents was limited.

## Supporting information

S1 Survey (PDF)
