# Supplementary material for: “People play it down and tell me it can’t kill people, but I know people are dying each day”. Children’s health literacy relating to a global pandemic (COVID-19); an international cross sectional study
Source: PLoS One. 2021 Feb 10;16(2):e0246405. doi: 10.1371/journal.pone.0246405 (PMC7875343; doi:10.1371/journal.pone.0246405)
Supplement: S1 Survey — (PDF) [file pone.0246405.s001.pdf]

Edge Hill University

The coronavirus information project - children's survey

Please read this information before starting the survey

Thank you for helping with our project. This survey is for children aged 7-12 living in the UK. Make sure you have checked with your mum/dad/carer that they are happy for you to answer this survey.

We are going to ask you to draw, label and send us a picture to explain 'why we are all trying to stay at home during the coronavirus'. You might want to do this drawing on a piece of paper before starting the survey.

Please answer as many questions as you can and tell us as much as possible, we are looking forward to seeing your pictures. There are no right or wrong answers, just tell us what you think. If you need help from your mum/dad/carer to fill out this survey that is okay.

If you click 'done' at the end of the survey then this means you are happy for us to use your answers as part of the project. Your answers are sent directly to the project team.

The lead for this project is Professor Lucy Bray, if you have any questions or concerns please ask your mum/dad/carer to contact us on brayl@edgehill.ac.uk

1. Where have <u>you</u> got information from about the coronavirus? (tick as many as you want)

- [ ] Mum/Dad/Carer
- [ ] School
- [ ] Friends
- [ ] YouTube
- [ ] TV
- [ ] Radio
- [ ] Newsround on CBBC
- [ ] Newspapers/magazines
- [ ] Internet (Google)
- [ ] NHS websites
- [ ] Social media (TikTok, Instagram, Facebook, Twitter)
- [ ] I haven't looked for any information

Other (type in other places you get information)

[ ]

2. From the choices above, which is the main way you get your information from about the coronavirus?

[ ]

3. Generally has the information been.....

- ◯ Very easy to understand
- ◯ Easy to understand
- ◯ Difficult to understand
- ◯ Very difficult to understand
- ◯ Not sure

4. How would you like to get information about the coronavirus ? (type in what you think)

5. Please tell us three things you know about coronavirus.....

1.

2.

3.

6. Please tell us about three things you would like to know about coronavirus....

1.

2.

3.

7. How much do you think you know about coronavirus?

- ◯ I know lots and lots
- ◯ I know quite a bit
- ◯ I know a little bit
- ◯ I don't know much

8. I find talking about the coronavirus:

- ☐ Interesting
- ☐ Worrying
- ☐ Fine
- ☐ Other (please type in)
- ☐ Boring
- ☐ Useful
- ☐ I don't talk about it

9. Please tell us 3 words you think of when thinking about the coronavirus:

1.

2.

3.

10. Could you draw and label a picture to explain '<u>why we are all trying to stay at home during the coronavirus</u>?'' (Ask your mum/dad carer to attach it to this survey by clicking on choose file and remember do not write your name on your picture).

Choose File Choose File No file chosen

Can you tell us a little bit about yourself (we will not be able to tell who you are from this information (check with your Mum/Dad/carer if you are not sure about this).

11. Where do you live?

- ◯ England
- ◯ Scotland
- ◯ Wales
- ◯ Ireland

12. How old are you?

- ◯ 7
- ◯ 8
- ◯ 9
- ◯ 10
- ◯ 11
- ◯ 12

13. How are you doing your school work at the moment?

- ◯ I am doing school work at home
- ◯ I am still going into school
- ◯ I am doing some school days at home and some school days going into school

Thank you so much for helping us and answering the questions.

If you are worried about coronavirus, then talk to your mum/dad or carer and there are places you can go for information and advice.
https://youngminds.org.uk/blog/what-to-do-if-you-re-anxious-about-coronavirus/
